# Low glucose dependent decrease of apoptosis and induction of autophagy in breast cancer MCF-7 cells

**DOI:** 10.1007/s11010-016-2711-4

**Published:** 2016-05-09

**Authors:** Rafał Krętowski, Małgorzata Borzym-Kluczyk, Anna Stypułkowska, Justyna Brańska-Januszewska, Halina Ostrowska, Marzanna Cechowska-Pasko

**Affiliations:** Department of Pharmaceutical Biochemistry, Medical University of Białystok, Mickiewicza 2A, 15-222 Białystok, Poland; Department of Biology, Medical University of Białystok, Białystok, Poland

**Keywords:** Apoptosis, Autophagy, CHOP, mTOR, NF-κB2, ORP150

## Abstract

Cancer cells have developed a number of adaptation mechanisms involving the signal activation of the transduction pathways, which promotes the progression and metastasis. Our results showed that the percentage of apoptotic MCF-7 cells incubated in the low glucose medium for 48 h was lower in comparison to those cultured in the high glucose medium, despite the high expression of the proapoptotic transcription factor—CHOP. Furthermore, the MCF-7 cells incubated in the low glucose medium for 48 h showed a higher expression of NF-κB p100/p52 subunits compared to cells incubated in the high glucose medium. Moreover, our findings demonstrated that the shortage of glucose strongly induces autophagy in MCF-7 cells. The activation of this process is not associated with the changes in the expression of mTOR kinase. We suggest, that the antiapoptotic chaperone ORP150 induction, transcription factor NF-κB2 activation, and increased autophagy constitute mechanisms protecting the MCF-7 cells against apoptosis.

## Introduction

Breast cancer is one of the most commonly diagnosed cancers in women worldwide. In addition, approximately one percent of men who have cancer suffer from breast cancer. Despite many breast cancer researches, there are no effective methods of fighting with this disease [1].

Glucose is the main energy source for tumor cells. They especially adapt in transporting extracellular glucose through the cell membrane into the cytoplasm, by up-regulation of glucose transporters—GLUT protein expression [2]. High level of intracellular glucose facilitates cancer cell proliferation because the metabolism of these cells is mainly glucose dependent. Changes in metabolic programing, from aerobic to anaerobic glycolysis are called the Warburg effect [3]. Under the shortage of oxygen, cancer cells can switch from full aerobic to a focus on less efficient but anaerobic glycolysis. Cancer cells usually obtain a substantial amount of energy from anaerobic glycolysis by converting most intracellular glucose to lactate rather than metabolizing it through oxidative phosphorylation. Despite the fact that ATP production by glycolysis can be more violent than by oxidative phosphorylation, the ATP generated is far less for per unit of glucose consumed [4]. Therefore, glucose is a crucial factor in cancer cell proliferation, regulating the expression of various proteins involved in apoptosis and autophagy [2].

Glucose deficiency disrupts the folding of newly synthesized proteins, and is one of the factors triggering the so-called endoplasmic reticulum stress (ER stress) [5]. In this process an accumulation of misfolded or unfolded proteins inside the endoplasmic reticulum is observed. Aggregation of damaged proteins in the ER, evokes unfolded protein response (UPR). The UPR process restoring ER homeostasis by activating a cascade of signaling molecules to global protein translation arrest and induction of biosynthesis of many molecular chaperones, including oxygen-regulated protein 150 (ORP150) and its glycosylated form glucose-regulated protein 170 (GRP170). It promotes the folding of hydrophobic regions in unfolded or misfolded polypeptides. The expression of ORP150 is upregulated under hypoxia, serum starvation, ischemia, and treatment cells with tunicamycin or 2-deoxyglucose. Moreover, ORP150 has been shown to generate antiapoptotic signals in certain tumors such as prostate, breast, and bladder cancer [6–8].

**Fig. 1:**
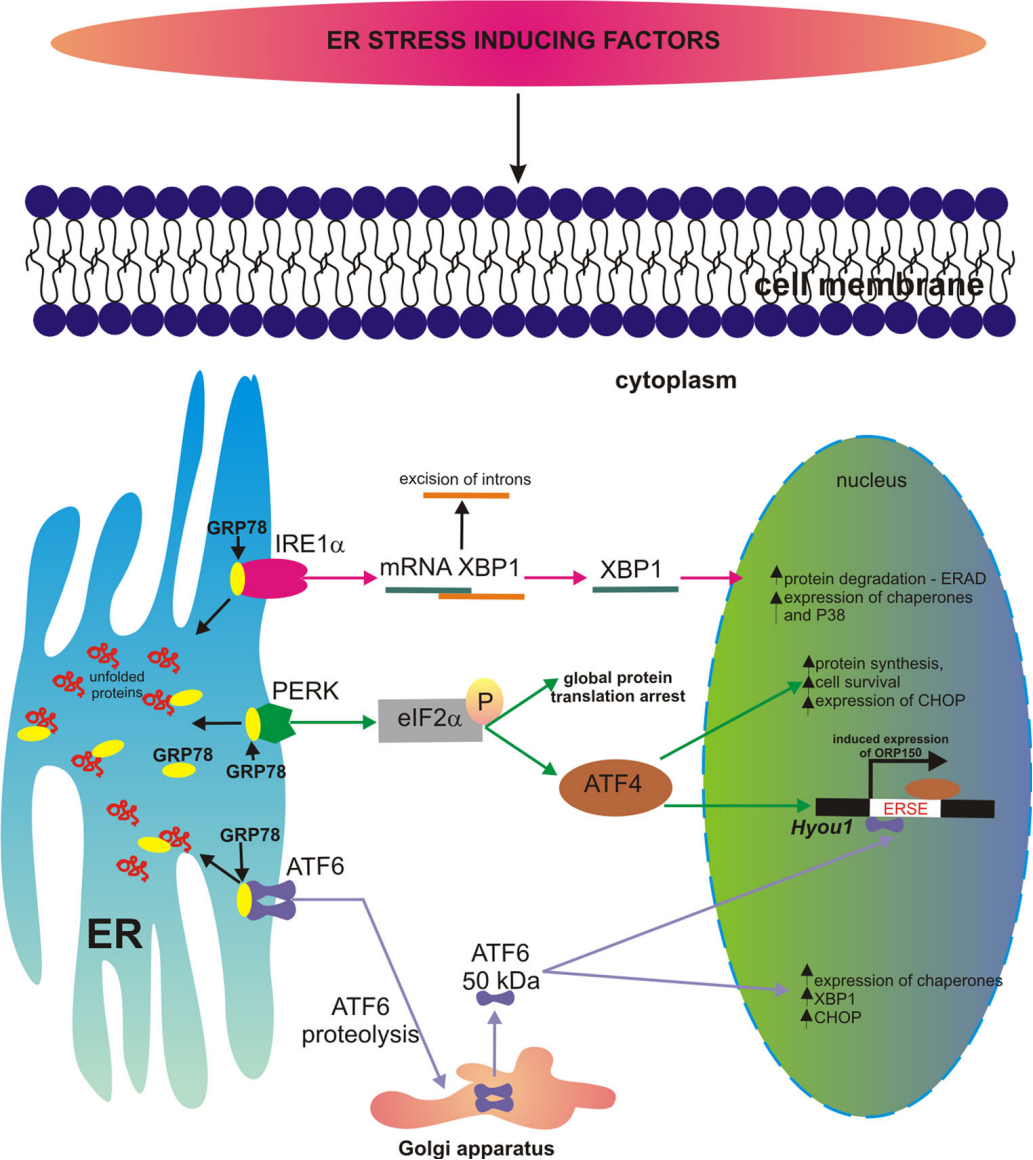
The mechanisms of transduction events associated with ER stress and UPR. Accumulation of unfolded or misfolded protein into endoplasmic reticulum three sensor proteins: IRE1α/β, PERK, and ATF6 are activated following their dissociation from ER chaperone GRP78. Activation of PERK leads to phosphorylation of eIF2a. Phosphorylated eIF2a specifically promotes the translation of the transcription factor ATF4 and induction of CHOP expression. Transcriptional factor CHOP is involved in proapoptotic signaling. ATF6 is activated by proteolysis mediated by proteases S1P and S2P. Next, both of the proteins are translocated from the ER to Golgi apparatus, where they become proteolytically cleaved to generate transcriptionally active proteins. The most important ATF6 target gene is X box binding protein 1 (XBP1), while IRE1 catalyzes the alternative splicing of XBP-1 mRNA leading to the expression of the active XBP1 transcription factor

Unfolded protein response (UPR) has three branches mediated by the following proteins: protein kinase like ER kinase (PERK), the inositol-requiring enzyme 1α (IRE1α), and the transcriptional factor-activating transcription factor 6 (ATF6), as was schematically presented in Fig. 1 [9]. All of these three sensor proteins have luminal domains, that bind to the ER chaperone GRP78, known as BIP, under unstressed conditions. Upon ER stress the GRP78 is released from these sensor proteins, permitting their oligomerization and thereby initiating the UPR to deal with accumulated unfolded proteins. The UPR response may activate the mechanisms that promote cancer growth and proliferation [5]. When the conformation of incorrectly folded proteins cannot be restored, the cell is directed to apoptosis by the increased expression of proapoptotic CCAAT enhancer binding protein homologous protein (CHOP). Induction of CHOP protein is a well-known apoptotic branch of ER stress. The CHOP plays a crucial role in the regulation of the expression of various genes involved in autophagy, cell cycle, or apoptosis [9].

The mechanism of apoptosis in MCF-7 cells is unclear and may be associated with mutations of the gene for caspase 3 [10]. There are no studies on the influence of glucose shortage on the mechanism of apoptosis and the role of a newly discovered chaperone protein ORP150 during ER stress conditions in MCF-7 cells.

An understanding of the mechanisms of apoptosis in breast cancer cells is important for clarifying of cancer pathogenesis and provides new possibilities in the treatment of breast cancer. Therefore, we decided to study the effect of glucose shortage on apoptosis, autophagy, and their mechanisms in breast cancer MCF-7 cell line.

## Materials and methods

### Reagents

Dulbecco’s modified Eagle’s medium (DMEM), containing glucose at 4.5 mg/ml (25 mM), Dulbecco’s modified Eagle’s medium (DMEM), containing glucose at 0.5 mg/ml (2.8 mM), l-glutamine, penicillin, streptomycin, trypsin–EDTA, FBS Gold, trypsin–EDTA were provided by Gibco (San Diego, USA), passive lysis buffer by Promega (Madison, USA), BCA Protein Assay Kit by Thermo Scientific (Rockford, USA), PE Annexin V Apoptosis Detection Kit I by BD Pharmingen™ (CA, USA), Sigma-Fast BCIP/NBT reagent, 4′,6-diamidino-2-phenylindole dihydrochloride—DAPI, donkey serum, acridine orange, ethidium bromide, camptothecin by Sigma (St Louis, MO, USA), monoclonal (mouse) anti-human ORP150 antibody by IBL (Gunma, Japan), medium coverquick by Hygeco (USA), polyclonal (rabbit) anti-human NF-κB2 p100/p52 antibody, monoclonal (rabbit) anti-human CHOP antibody, monoclonal (rabbit) anti-human β-tubulin antibody, alkaline phosphatase-labeled anti-rabbit immunoglobulin G were provided by Cell Signaling Technology (Boston, USA), monoclonal (mouse) anti-human mTOR antibody (Merck-Millipore, USA), alkaline phosphatase-labeled anti-mouse immunoglobulin G by Rockland (PA, USA), polyclonal (rabbit) anti-human LC3 by Abgen, donkey anti-rabbit IgG conjugated with Alexa Fluor 488 by Molecular Probes (USA).

### Cell cultures

Human breast cancer cell line MCF-7, which are estrogen receptor positive, were obtained from American Type Culture Collection (ATCC). Cells were maintained in high glucose DMEM, supplemented with 10 % heat-inactivated fetal bovine serum GOLD (FBS GOLD), penicillin (100 U/mL), and streptomycin (100 μg/mL). Cells were cultured in Falcon flasks (BD) in a 5 % CO_2_ incubator (Galaxy S+; New Brunswick), at 37 °C. Cells were grown to confluence and passaged with 0.05 % trypsin, 0.02 % EDTA in calcium-free phosphate-buffered saline (PBS), and counted in a Scepter cell counter (Millipore), then 2.5 × 10^5^ cells were seeded in six-well plates in 2 ml of growth medium. In these conditions they reached 70 % of confluence. The following medium of various glucose concentrations were used: the high glucose DMEM contained 4.5 mg of glucose per ml (25 mM) or the low glucose DMEM contained 0.5 mg of glucose per ml (2.8 mM) supplemented with 10 % heat-inactivated fetal bovine serum GOLD (FBS GOLD), 2 mM l-glutamine, penicillin (100 U/ml), and streptomycin (100 μg/ml). The incubation was continued for 12, 24, and 48 h. After incubation the culture media were removed, the cell layers were washed with PBS and submitted to the action of lysis buffer for the determination of protein concentration or trypsinized to determinate apoptosis and necrosis by flow cytometry method.

### Detection of apoptosis

Cells were incubated in the high glucose or low glucose DMEM for 12, 24, and 48 h. Apoptosis was evaluated by flow cytometry on FACSCanto II cytometer (Becton-Dickinson). The cells were trypsinised and resuspended in DMEM. After that time, the cells were suspended in binding buffer for staining with FITC-Annexin V and propidium iodide—PI for 15 min at room temperature in the dark following the manufacturer’s instructions (FITC Annexin V apoptosis detection Kit I). The signal obtained from cells stained with annexin V or PI alone was used for fluorescence compensation. Data were analyzed with FACSDiva software.

### Fluorescent microscopy assay

Staining cells with fluorescent dyes, including acridine orange and ethidium bromide, is used in the evaluation of the nuclear morphology of apoptotic and necrotic cells. The MCF-7 cells grew on cover glass with high glucose or low glucose medium for 12, 24, and 48 h. After these times, the cells were washed twice with PBS and stained with 1 ml of the dye mixture (10 μM acridine orange and 10 μM ethidium bromide in PBS) for 10 min in dark at room temperature. Next, the stained solution was removed and the cell layers were washed with PBS, analyzed, and photographed under a fluorescence microscope at 200-fold magnification. Acridine orange is a vital dye that will stain both live and dead cells, whereas ethidium bromide will stain only those cells that have lost their membrane integrity. The hundred cells per sample were analyzed by fluorescence microscope (Olympus CXK41, U-RLFT50) according to the following criteria: living cells—normal green nucleus; early apoptotic cells—bright green nucleus with condensed or fragmentated chromatin; late apoptotic cells—orange-stained nuclei with chromatin condensation or fragmentation while necrotic cells were characterized by orange-stained cell nuclei. Percentage of apoptotic cells was the sum of early apoptotic and late apoptotic cell percent. Optimal parameter settings were found using a positive control: the MCF-7 cells were incubated with 10 µM camptothecin in high glucose DMEM in a 5 % CO_2_ incubator (Galaxy S+; New Brunswick), at 37 °C for 12 h. After that time the cells were analyzed under fluorescence microscopy.

### Sodium dodecyl sulfate/polyacrylamide gel electrophoresis (SDS/PAGE)

Cells were washed with cold PBS and solubilized in 200 μl of passive lysis buffer per well. The lysates were centrifuged for 15 min, at 12,000×*g*, at 4 °C. Samples of lysates containing 30 μg of protein were subjected to SDS-PAGE, as described by Laemmli [11]. The electrophoresis runs for 40–45 min. In each experiment 7.5 % polyacrylamide gel and constant current (25 mA) were used.

### Immunoblotting

The proteins were transferred to nitrocellulose membranes and then pre-treated for 2 h with Tris-buffered saline (TBS) containing 0.05 % Tween 20 (TBS-T) and 5 % non-fat dry milk, at room temperature. Membranes were probed for 16 h with a mixture containing monoclonal (mouse) anti-human ORP150 antibody (1:100), polyclonal (rabbit) anti-human NF-κB2 p100/p52 antibody (1:1000), monoclonal (mouse) anti-human CHOP (1:1000), polyclonal (rabbit) anti-human β-tubulin antibody (1:1000), monoclonal (mouse) anti-human mTOR in 5 % dried milk in TBS-T, at 4 °C. Then the alkaline phosphatase conjugated antibody against mouse or rabbit IgG (whole molecule) at a 1:2500 dilution in TBS-T was added for 1 h in TBS-T with slow shaking. The nitrocellulose was washed with TBS-T (five times for 5 min) and exposed to Sigma-Fast BCIP/NBT reagent.

### Immunofluorescence

The MCF7 cells grown on cover glass with high or low glucose medium for 12, 24, and 48 h. After these times, they were washed with cold PBS and then fixed in 4 % paraformaldehyde in PBS for 10 min at room temperature. After fixation, the cells were permeabilized in PBS containing 0.2 % Triton-X100 for 5 min and blocked in 5 % normal donkey serum at room temperature for 60 min to block non-specific reactions. Then cells were incubated with polyclonal (rabbit) anti-human anti-LC3 antibody (1:500) for 60 min at room temperature. After incubation, they were washed further three times in PBS and incubated in donkey anti-rabbit IgG conjugated with Alexa Fluor 488 (1:200) at room temperature for 1 h. Then, cells were washed three times in PBS and stained with 4′,6′-diamidino-2-phenylindole—DAPI for 10 min to indicate the nucleus. Samples were washed twice in PBS and mounted on microscopy slides in medium coverquick, dried overnight, and stored in the dark until viewing. Immunolabeled cells were analyzed by using camera Nikon Digital Sight DS-Fi1 and a fluorescence microscope Nikon ECLIPSE Ti/C1 Plus, equipped with two filters DAPI (blue) and FITC (green), (excitation wavelength/emission filter: 405/450 nm, 488/515 nm, respectively) at 200-fold magnification. The hundred cells per sample were examined by fluorescence microscopy, according to the following criteria: the LC3-positive cells were determined by counting the number of cells with green signal from anti-LC3 staining in cytoplasm and DAPI blue fluorescence nucleus comparison to the number of cells with only DAPI fluorescence. Optimal parameter settings were found using an LC3-positive control: the MCF-7 cells were incubated in PBS in a 5 % CO_2_ incubator (Galaxy S+; New Brunswick), at 37 °C for 2 h. After that time the cells were analyzed under fluorescence microscopy.

### Protein assay

Protein concentration in cell lysates was determined by the method of Smith [12] using BCA Protein Assay Kit (Thermo Scientific, USA). Bovine serum albumin was used as a standard.

### Statistical analysis

Mean values from three independent experiments ± standard deviations (SD) were calculated. The data were statistically analyzed using one way-ANOVA followed by Tukey’s post Hoc *t*-test analysis. The significant differences of means was determined at the level of *p < 0.05 or **p < 0.001.

## Results

### The effect of glucose shortage on the expression of CHOP

**Fig. 2:**
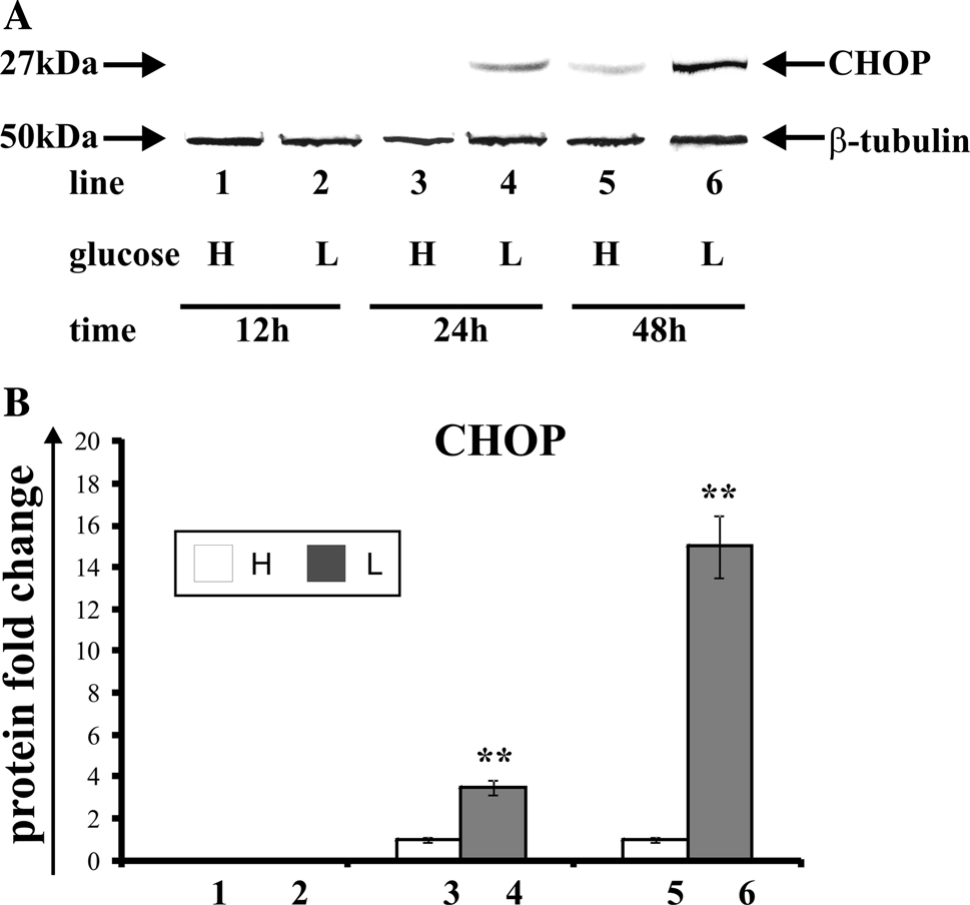
Western blot (**a**) and densitometric analysis (**b**) of CHOP expression in MCF-7 cells incubated in high glucose (H) or low glucose (L) DMEM for 12, 24, and 48 h. Samples containing 30 μg of protein were submitted to electrophoresis and immunoblotting. A representative Western blot is presented (**a**). Densitometric analysis was presented as relative protein expression. The expression of β-tublin served as a control for protein loading. Mean values of densitometric analysis from three independent experiments ± SD are presented (**b**). **p < 0.001

Transcription factor CHOP participates in both apoptosis and the impediment of cell growth. Also, it is known as the marker of endoplasmic stress. Figure 2 shows the effect of glucose shortage on the expression of transcription factor CHOP in breast cancer MCF-7 cell line (Fig. 2a—Western blot analysis, Fig. 2b—densitometric analysis). The cells were incubated for 12, 24, and 48 h in high glucose (H), (Fig. 2, line: 1, 3, 5) or low glucose (L) medium (Fig. 2, line: 2, 4, 6). The expression of CHOP depends on glucose concentration in medium and on the time of incubation. The MCF-7 cells incubated in high glucose and low glucose medium for 12 h did not express the transcription factor CHOP (Fig. 2, line: 1, 2). The incubation of these cells in low glucose medium for 24 h resulted in the appearance of band corresponding to CHOP (Fig. 2, line: 4), whereas the cells incubated in high glucose medium (Fig. 2, line: 3) did not express this protein. Prolongation of incubation time up to 48 h in the cells incubated in low glucose medium resulted in a fourfold increase of CHOP expression (Fig. 2, line: 6) in comparison to the cells incubated in high glucose medium (Fig. 2, line: 5).

### The effect of glucose shortage on apoptosis and necrosis

**Fig. 3:**
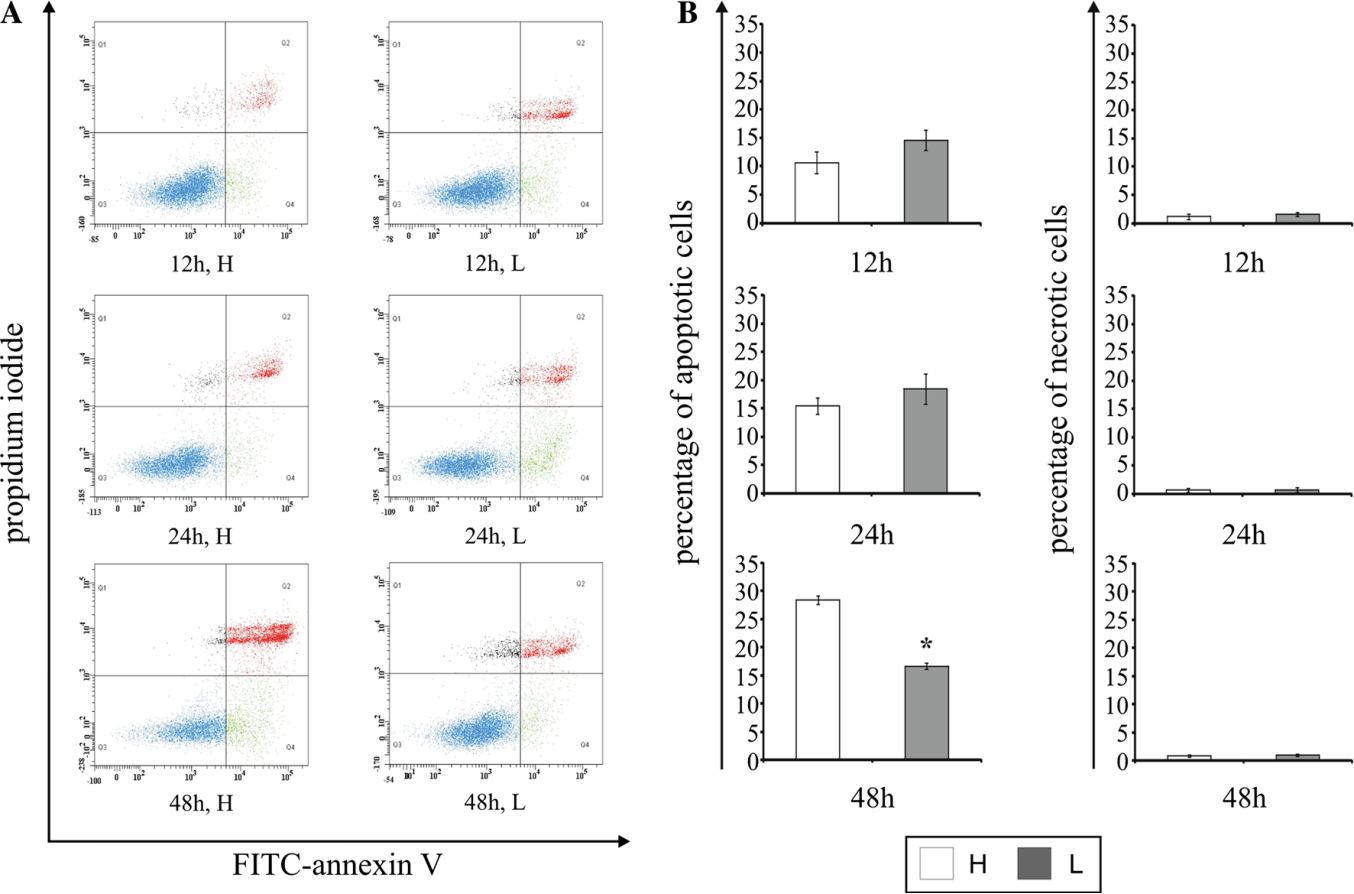
The effect of glucose shortage on apoptosis and necrosis in breast cancer MCF-7 cell line evaluated by annexin V assay. The cells were incubated for 12, 24, and 48 h in high glucose (H) or low glucose (L) DMEM and double-stained FITC-Annexin V/PI. Following acquisition of sample, the cells were gated thought the FSC and SSC and analyzed for fluorescence intensity of FITC-Annexin V and propidium iodide (PI), (A). The MCF-7 cells were considered into four subpopulations: live cells—Q3 (annexin V-FITC^−^/PI^−^), early apoptotic cells—Q4 (annexin V-FITC^+^/PI^−^), late apoptotic cells—Q2 (annexin V-FITC^+^/PI^+^), and necrotic cells (annexin V-FITC^−^/PI^+^)—Q1 (**a**). Percentage of apoptotic cells was the sum percentage of early apoptotic (Q4) and late apoptotic cells (Q2). Mean values from three independent experiments ± SD are presented (**b**). *p < 0.05

An apoptosis of MCF-7 cell was evaluated by flow cytometry on FACSCanto II cytometer (Becton-Dickinson). Figure 3b shows the percent of apoptotic and necrotic cells in cultures incubated for 12, 24, and 48 h in high glucose (H) and low glucose (L) medium. Figure 3a shows representative histograms of MCF-7 cells FACS analysis via Annexin V-FITC/PI staining. We did not observe significant changes between the cells incubated for 12 and 24 h in high and low glucose medium. Prolongation of incubation time up to 48 h in high glucose medium resulted in increase of apoptosis up to 28 %. Interesting is the fact that, after 48 h of MCF-7 incubation in low glucose medium, the percentage of apoptotic cells was lower (approximately 43 %) compared to the culture growing in the high glucose medium. Furthermore, the percentage of necrotic cells did not change significantly, notwithstanding on incubation time and glucose concentration in the medium (Fig. 3a, b).

**Fig. 4:**
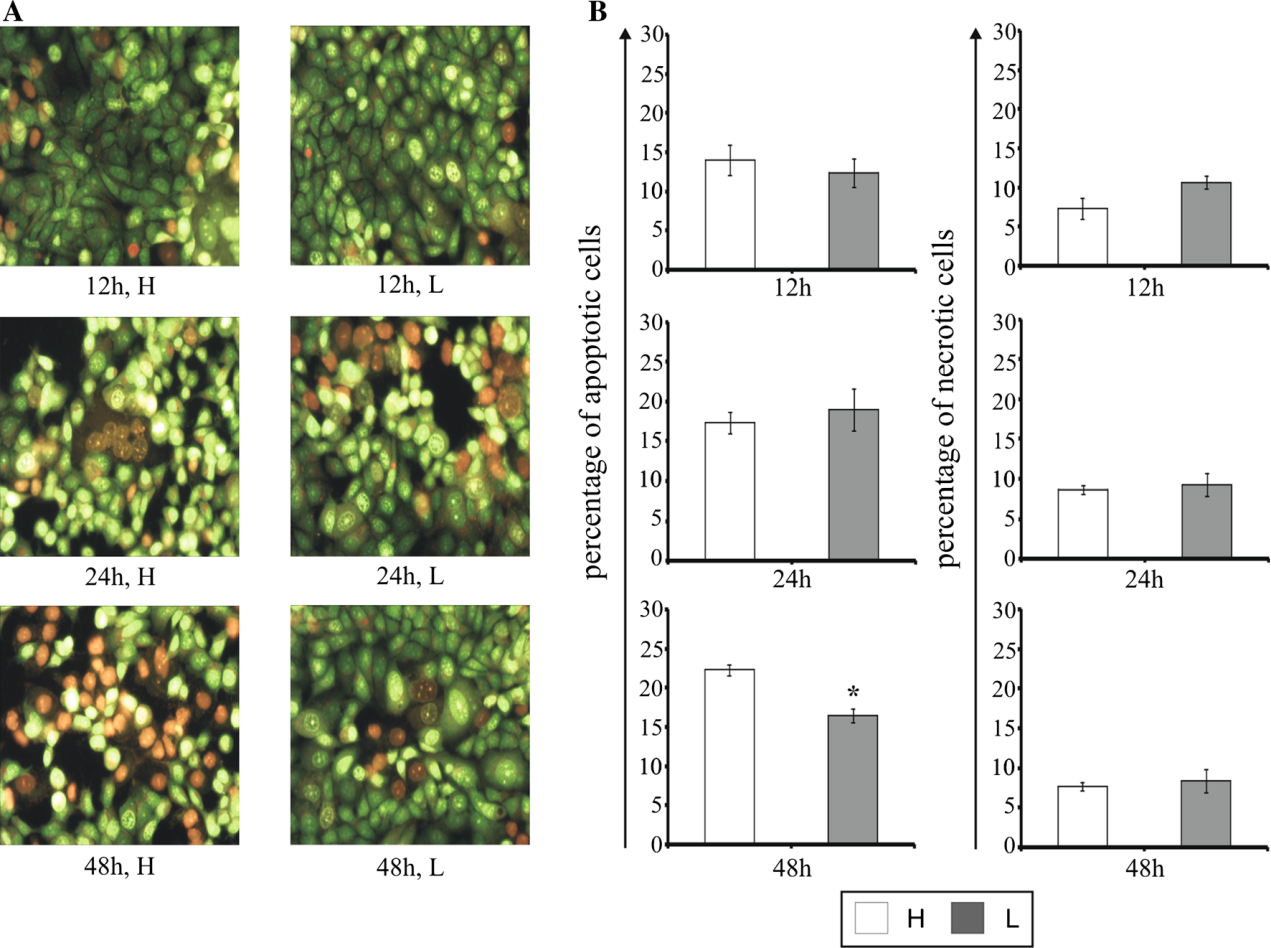
The effect glucose shortage on apoptosis and necrosis in the breast cancer MCF-7 cell line evaluated by fluorescence microscope assay. The cells were incubated in high glucose (H) or low glucose (L) DMEM for 12, 24, and 48 h and stained with acridine orange and ethidium bromide. The cells were photographed under a fluorescence microscope at 200-fold magnification and analyzed according to the following criteria: living cells, early apoptotic cells, late apoptotic cells, and necrotic cells. We presented representative images form one of three independent experiments (**a**). Percentage of apoptotic cells was the sum percentage of early apoptotic and late apoptotic cells (**b**). Mean values from three independent experiments ± SD are presented. *p < 0.05

Staining cells with fluorescent dyes, including acridine orange and ethidium bromide, was used for the evaluation of the apoptotic and necrotic cells morphology (Fig. 4a). Figure 4b shows the percent of apoptotic and necrotic cells incubated in high glucose and low glucose medium for 12, 24, and, 48 h. Similar to the flow cytometry analysis, we did not observe significant changes between the cells incubated for 12 and 24 h in high glucose and low glucose medium. Prolongation of incubation time up to 48 h in low glucose medium demonstrated 30 % reduction of apoptosis in comparison to cells maintained in high glucose medium. Furthermore, the percentage of necrotic cells did not change significantly; notwithstanding on incubation time and glucose concentration in the medium but it was higher than in the flow cytometry analysis.

### The effect of glucose shortage on the expression of chaperone protein ORP150

**Fig. 5:**
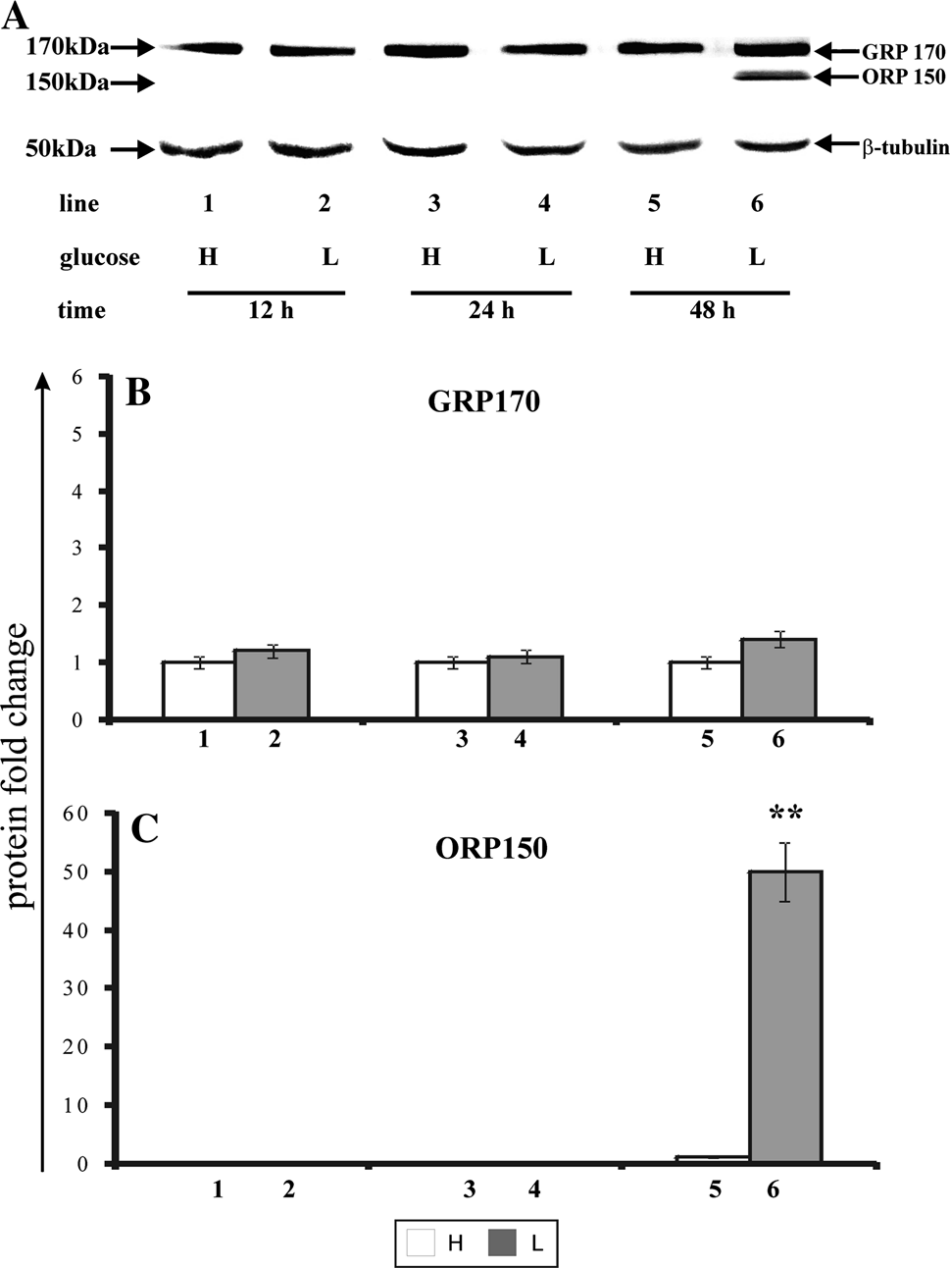
Western blot (**a**) and densitometric analysis and of GRP170 (**b**) and ORP150 (**c**) expression in MCF-7 cells incubated in high glucose (H) and low glucose (L) DMEM for 12, 24, and 48 h. Samples containing 30 μg of protein were submitted to electrophoresis and immunoblotting. A representative Western blot is presented. Densitometric analysis was presented as relative protein expression. The expression of β-tublin served as a control for protein loading. Mean values of densitometric analysis from three independent experiments ± SD are presented. **p < 0.001

During ER stress the cancer cells activate the unfolded protein response (UPR), which restores the intracellular homeostasis through the induction of a number of endoplasmic reticulum chaperones. One of the recently found proteins, belonging to the GRP family, is ORP150 (oxygen-regulated protein 150). Figure 5 shows the effect of glucose shortage on the expression of ORP150 and its glycosylated form—GRP170 in MCF-7 cell line (Fig. 5a—Western blot analysis, Fig. 5b, c—densitometric analysis). The cells were incubated for 12, 24, and 48 h in high glucose (H), (Fig. 5, line: 1, 3, 5) or low glucose (L) medium (Fig. 5, line: 2, 4, 6). The expression of GRP170 observed in cultures incubated in high glucose and low glucose medium, was notwithstanding on incubation time (Fig. 5a, b, lanes: 1–6). In contrast, the expression of ORP150 was dependent on both the glucose concentration in medium and on the time of incubation (Fig. 5a, b, lines 6). While, the cells incubated in high glucose and low glucose medium for 12 h or 24 h did not express chaperone protein ORP150 (Fig. 5a, c, lanes: 1–4), the prolongation of incubation time up to 48 h in low glucose medium resulted in an appearance of a band corresponding to molecular mass of ORP150 (Fig. 5a, c, lane 6).

### The effect of glucose shortage on the expression of transcriptional factor NF-κB2

**Fig. 6:**
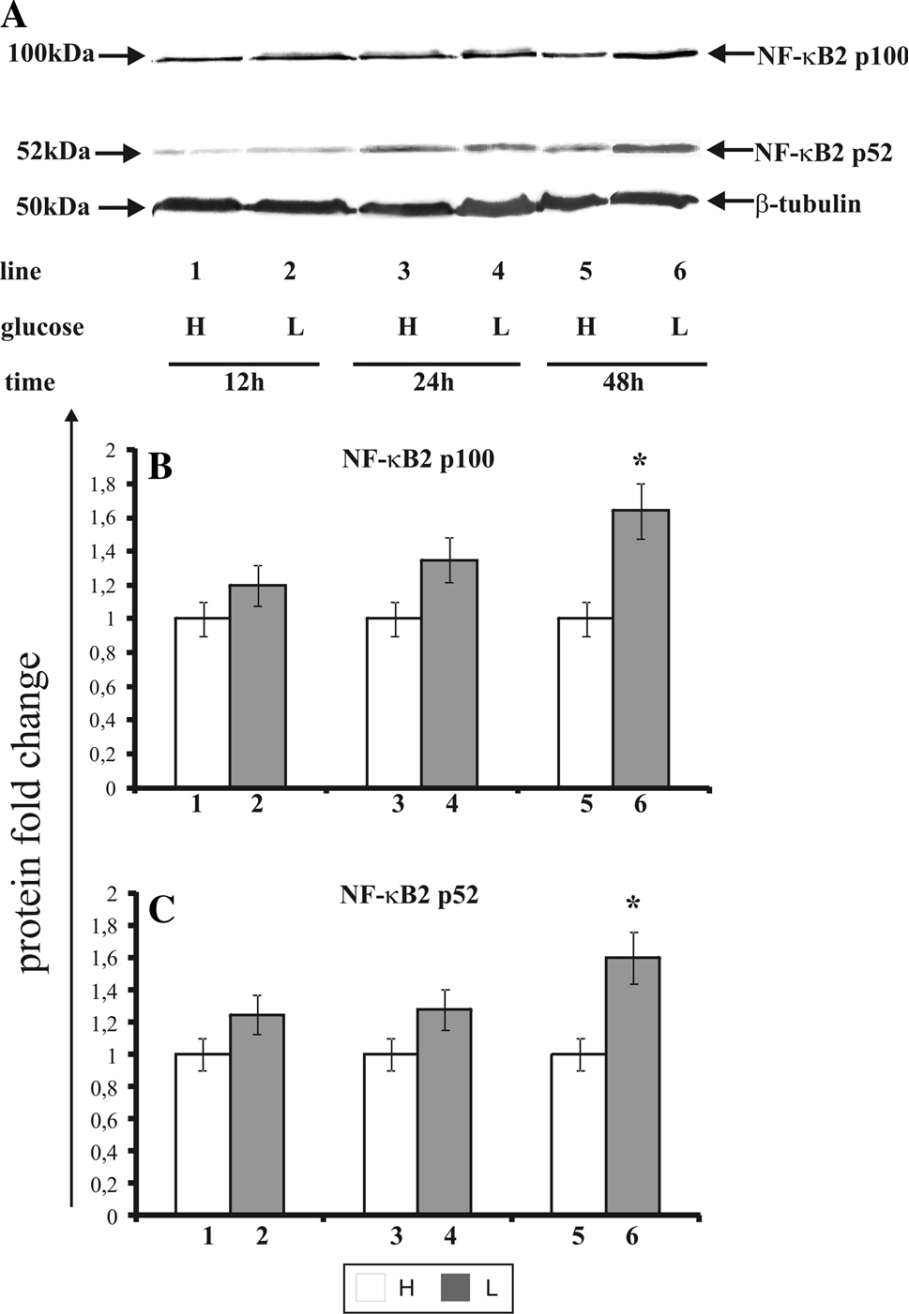
Western blot (**a**) and densitometric analysis of NF-κB2 p100 (**b**) and NF-κB2 p52 (**c**) subunits expression in MCF-7 cells incubated in high glucose (H) and low glucose (L) DMEM for 12, 24, and 48 h. Samples containing 30 μg of protein were submitted to electrophoresis and immunoblotting. A representative Western blot is presented. Densitometric analysis was presented as relative protein expression. The expression of β-tublin served as a control for protein loading. Mean values of densitometric analysis from three independent experiments ± SD are presented (**b**, **c**). **p < 0.001 or *p < 0.05

The activation of NF-κB2 takes place during endoplasmic reticulum stress. It involves generation of the 100 kDa molecular weight (p100 subunit) and a transcriptionally active form of this protein of the 52 kDa molecular weight (p52 subunit). This protein undergoes translocation to cell nucleus, binds with a particular DNA region and thus either enhances or impedes the synthesis of the proteins vital for cell function. Figure 6 shows the effect of glucose shortage on the expression of NF-κB2 in MCF-7 cell line (Fig. 6a—Western blot analysis, Fig. 6b, c—densitometric analysis). The cells were incubated for 12, 24, and 48 h in high glucose (H), (Fig. 6, line: 1, 3, 5) or low glucose (L) medium (Fig. 6, line: 2, 4, 6). Interestingly, the expression of NF-κB2 transcription factor was dependent on glucose concentration in the medium. There was no statistically significant differences in the expression levels of NF-κB2 in MCF-7 cells incubated for 12 and 24 h in low glucose medium, (Fig. 6, line: 2, 4) compared to cells incubated in high glucose medium, (Fig. 6, line: 1, 3), while the cells incubated for 48 h in the low glucose medium show a higher expression of p100 subunit (approximately 30 %) and p52 subunit (approximately 35 %) compared to the cells incubated in a high glucose medium.

### The effect of glucose shortage on the expression of LC3

**Fig. 7:**
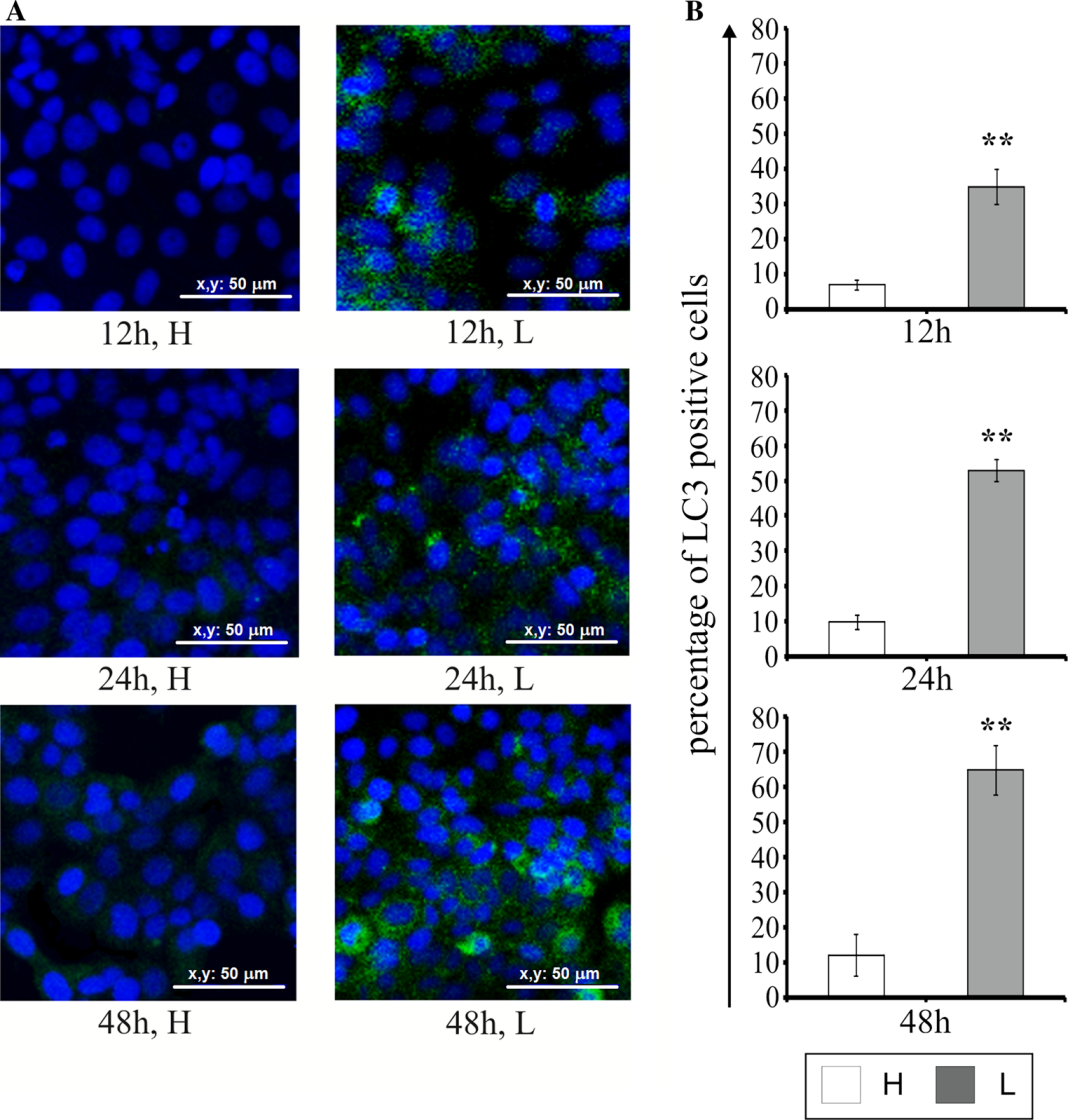
Detection of the autophagy marker, LC3 by immunofluorescence in MCF-7 cells incubated in high glucose (H) or low glucose (L) medium for 12, 24, and 48 h (**a**) and the percentage of LC3-positive cells (**b**). The nuclei are stained in blue (DAPI staining method). Mean values from three independent experiments ± SD are presented (**b**). **p < 0.001. *Scale bar* 50 μm

The LC3 protein is known marker of autophagy. The effect of glucose shortage on LC3 expression in MCF-7 cells was evaluated by immunofluorescence, and the results are shown in Fig. 7. Only a few of LC3-positive cells were observed in cells incubated with high glucose for 12–48 h (Fig. 7a, b). Decreased glucose concentration resulted in the accumulation of the LC3 protein in a time-dependent manner (Fig. 7a, b). It is worthy of note that after 12 h of incubation in low glucose medium, the percentage of cells with LC3 expression was approximately five-fold higher than in the cells incubated in high glucose medium (Fig. 7b). The most intense fluorescence of the antibody-labeled LC3 protein was observed after 48 h of MCF-7 cells incubation in the low glucose medium. We also noticed the accumulation of LC3 protein around the nucleus (Fig. 7a), which may indicate the formation of autophagosomes.

### The effect of glucose shortage on the mTOR kinase expression

**Fig. 8:**
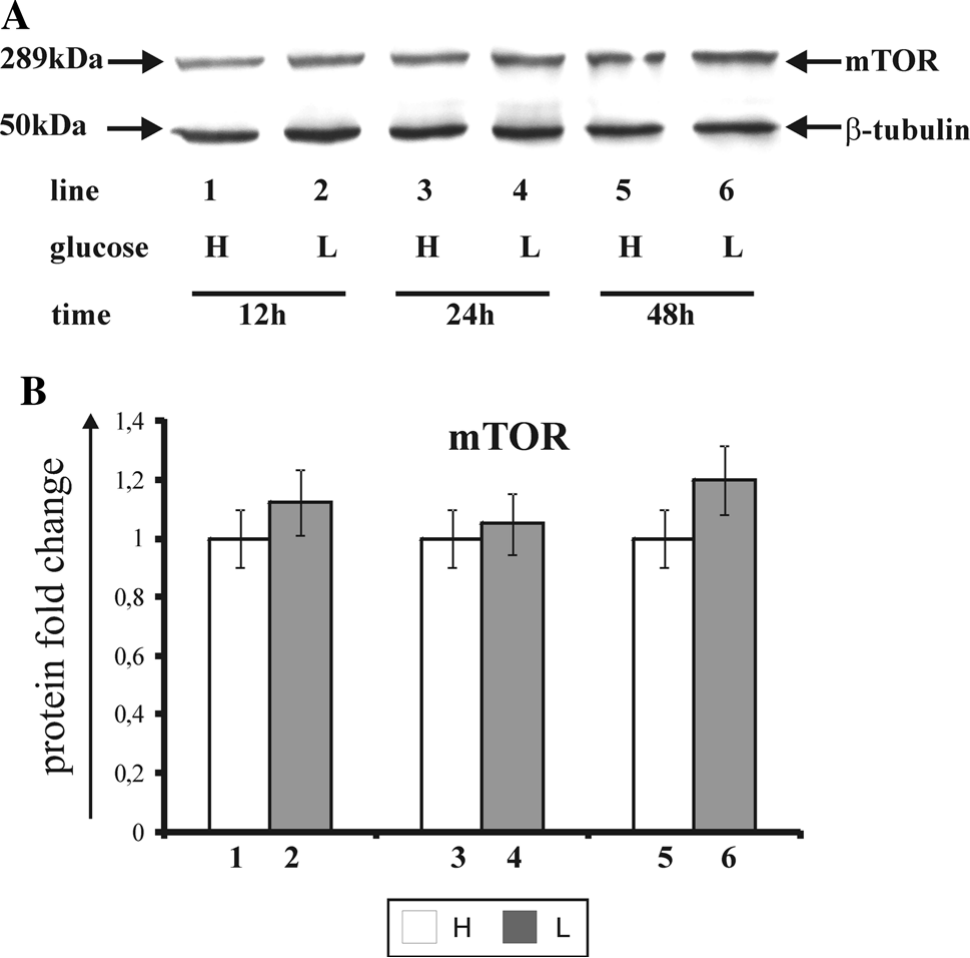
Western blot (**a**) and densitometric analysis (**b**) of mTOR kinase expression in MCF-7 cells incubated in high glucose (H) and low glucose (L) DMEM for 12, 24, and 48 h. Samples containing 30 μg of protein were submitted to electrophoresis and immunoblotting. Densitometric analysis was presented as relative protein expression. The expression of β-tublin served as a control for protein loading. A representative Western blot from one of three independent experiments is presented (**a**). Mean values of densitometric analysis from three independent experiments ± SD are presented (**b**)

The mammalian target of rapamycin (mTOR) controls the cell growth in response to nutrients and growth factors and is frequently deregulated in cancer. Figure 8 shows the effect of glucose shortage on mTOR kinase expression in breast cancer MCF-7 cell line (Fig. 8a—Western blot analysis, Fig. 8b—densitometric analysis). The cells were incubated for 12, 24, and 48 h in high (H), (Fig. 8, line: 1, 3, 5) and low glucose (L) medium (Fig. 8, line: 2, 4, 6). The mTOR kinase expression was notwithstanding on the time and glucose concentration in the medium (Fig. 8, line: 1–6). We did not observe significant changes between the cells incubated for 12, 24, and 48 h in high glucose and low glucose medium.

## Discussion

Shortage of nutrients such as glucose as well as oxygen deficiency, oxidative stress, disturbances in the homeostasis of calcium ions, and protein glycosylation increase the intracellular protein aggregation [13, 14]. Accumulation of the unfolded proteins and protein aggregates impair the function of the endoplasmic reticulum and thus leads to both the endoplasmic stress and the activation of the signal transduction pathways associated with the cellular unfolded protein response. The UPR process is aimed at restoring the intracellular homeostasis by the induction of the adaptation pathways related to the activation of three transmembrane proteins of the endoplasmic reticulum: IRE1, PERK, and ATF6. They participate in the process of enhanced expression of chaperones, among others ORP150, GRP78, GRP94, and transcription factors, among others: XBP1, CHOP, NF-κB, antioxidative protection, autophagy, or the activation of translational block [15–17]. Thus the UPR, while activated as a pro-survival response under moderate or intermittent ER stress, can also lead to other under conditions of acute or chronic stress [5].

Apoptosis is a complex and multi-stage process. During this process a variety of biochemical and morphological changes take place through different signal transduction pathways. Activation of one of the pathways is associated with the endoplasmic reticulum stress induced by glucose shortage [18, 19]. A long-lasting ER stress or damage quality control system of newly synthesized proteins in MCF-7 cells results in an increased expression of the proapoptotic transcription factor CHOP that promotes apoptosis [20]. According to our results, CHOP expression depends on both glucose concentration in the medium and on the time of incubation. We observed that the expression of proapoptotic factor—CHOP was strongly increased in the MCF-7 cells incubated in low glucose medium in contrast to these cells incubated in high glucose medium.

CHOP transcription factor participates in both apoptosis and in the arrest of cell growth. Also, it is the marker of endoplasmic stress. In physiological conditions its expression is very low [20–22]. ER stress activates the transduction pathways of the signals dependent on the three sensor proteins: IRE1, PERK, and ATF6. The major effect of PERK-eIF2α-ATF4 and ATF6 activation is the arrest of protein synthesis due to the inhibition of mRNA transcription, to the activation of ATF4 and ATF6 transcription factors and to the increase of the CHOP expression [23]. Increased CHOP synthesis promotes the mitochondrial apoptotic pathway in numerous cancer cell lines. The induction of the transcription activator—CHOP may enhance the synthesis of other proapoptotic proteins such as DR5, GADD34, and TRB3. Moreover, it has been observed that the cells that lack the expression of CHOP^-/−^ transcription factor show resistance to the apoptosis induced by chemotherapeutic effect [24].

The mechanism of apoptosis by the induction of CHOP transcription factor expression involves the decrease in the synthesis of the BCL-2 antiapoptotic proteins and the increase in the expression of the proapoptotic proteins: BIM, PUMA, and BAX [25]. It is known that BIM protein increases apoptosis by binding and neutralizing certain antiapoptotic proteins such as BCL-2 and BCL-xL. Proapoptotic proteins contain α-helical domains, which participate in the formation of channels in mitochondrial membrane. The proapoptotic protein BAX interacts with megachannels, also known as permeability transition pore, increases the permeability of mitochondrial membrane for cytochrome c. Cytochrome c and APAF-1 proteins form the complex, which activates procaspase 9. Active caspase 9 leads to the activation of executive caspases, which are directly responsible for cell death [19, 26].

According to our results, the percentage of apoptotic MCF-7 cells incubated in the low glucose medium was lower compared to the culture grown in high glucose medium, despite increased expression of the CHOP proapoptotic transcription factor.

During cancer growth, cells are at risk of unfavorable conditions in the tumor microenvironment: nutrient deficiency, oxidative stress, lack of growth factors, and hypoxia. Cancer cells have developed a number of adaptation mechanisms involving the activation of the transduction pathways of the signals, which promote the growth progression and metastasis [27]. These study indicate that mammary cancer cells adapt to ER stress and thus activate a number of processes which promote proliferation such as: expression of the antiapoptotic chaperone ORP150, increase in the expression of NF-κB2 transcription factor as well as increased autophagy.

It is of interest, that MCF-7 breast cancer cell line do not express executor caspase 3, also known as CPP32, Yama, or apopain, as result of a 47 kb deletion in exone 3 of the *CPP32* gene [10]. Despite this, MCF-7 cells undergo apoptosis after treatment with anti-cancer drugs. This suggests the presence of an alternative enhancement pathways. Initiator caspases, include caspases 8 or 9, can lead to apoptosis enhancement through activation of caspase 3 not only, but also activation of caspase 7. *Talatian at al.* showed that, decreased expression of caspase 7 protected the MCF-7 cells from the DNA-cleaving antimitotic agent, induced apoptosis—neocarzinostatin. Caspase 7 is highly related to caspases 3 and show the same synthetic substrate specificity in vitro [28].

One of the mechanisms that explains the impediment of apoptosis in MCF-7 cells in ER stress, caused by glucose shortage, is the enhanced expression of antiapoptotic chaperone ORP150. The family of HSP70 proteins, which includes ORP150, plays a crucial role in maintaining the integrity of mitochondrial membrane and thus participates in the control of cytochrome c release to cell cytoplasm. This protein acts with APAF-1 factor and prevents its oligomerization and next blocks its interaction with procaspase 9 [29]. During apoptosis the AIF—apoptosis-inducing factor is released from mitochondria and HSP70 complex is formed. This way the AIF translocation to cell nucleus is blocked and the DNA degradation is impeded [20]. Increased expression of HSP70 chaperone is observed in various malignant tumors: mammary cancer, kidney, and urinary bladder [5].

Cechowska-Pasko et al. showed that in endoplasmic stress caused by low glucose concentration in the medium, the induction of ORP150 expression and the apoptosis impediment in HeLa cells from cervical cancer take place. Transfection of HeLa cells with a specific to ORP150 siRNA led to an increased apoptosis [30]. Our study confirms that the antiapoptotic effect of ORP150 chaperone in ER stress is caused by glucose shortage.

Cancer cells have developed a number of signal transduction pathways responsible for the regulation of the expression of various genes [31]. Due to the fact that nuclear factor NF-κB participates in cell response to unfolded proteins, it was decided to evaluate its expression in MCF-7 cells of mammary cancer in ER stress caused by low glucose concentration in the medium. NF-κB2 is a transcription factor that regulates apoptosis, cell proliferation, arrest of cell cycle, and angiogenesis [32]. During ER stress the proteolytic activation of this factor takes place. It involves generation of a subunit of the mass 100 kDa (p100) and a transcriptionally active form of this protein of a molecular weight 52 kDa (p52). This protein undergoes translocation to cell nucleus, binds with a particular DNA region, and thus either enhances or impedes the synthesis of the proteins that are vital for cell function. The expression of NF-κB2 transcription factor depends on glucose concentration in the medium. MCF-7 cells incubated for 48 h in the low glucose medium showed a higher expression of p100 subunit and p52 subunit compared to the cells incubated in a high glucose medium. We suggest, that NF-κB2 transcription factor enhances cancer cell proliferation and promotes the expression of antiapoptotic proteins from BCL-2 family [33].

The UPR process limits the translation of numerous proteins including the IκB inhibitor protein responsible for NF-κB2 retention in cell cytoplasm. The IκB molecule is phosphorylated by IKK protein kinase and next undergoes degradation within proteasomes. NF-κB2 shows a characteristic property of translocating to cell nucleus and activating or impeding the transcription of a number of genes [34]. NF-κB2 transcription factor activates the synthesis of the proteins that play the role of IAP caspase inhibitors such as c-IAP1/2, XIAP, as well as antiapoptotic proteins: BCL-xL and BLF-1. Moreover, increased expression of NF-κB2 correlates with cancer cell resistance to apoptosis by the progression of cell cycle and the development of the resistance to chemotherapeutic agents [35].

In case of low glucose, cancer cells use the energy stored in their own cellular structures [36]. We decided to study the effect of glucose shortage on the autophagy in the breast cancer MCF-7 cell line. In order to assess the autophagy in MCF-7 cells the immunodetection of LC3 protein autophagy marker was performed with fluorescence microscopy. It was observed that the LC3 expression depends on glucose concentration in the medium and the duration of cell incubation. Decreased glucose concentration contributes to the enhancement of accumulation of the LC3 protein labeled with fluorochrome in the autophagosome membrane.

Autophagy is a multi-stage catabolic process necessary to maintain intracellular homoeostasis [37]. It is a physiological process of lysosomal degradation that, in response to environmental stresses, may either promote cell survival or death depending on many factors. Increased autophagy is most commonly caused by the deficiency of nutrients including glucose, amino acids, growth factors, low energetic level of the cell, endoplasmic reticulum stress, oxidative stress, hypoxia, or damage to cellular organelle. Three different autophagy pathways can be found: macroautophagy, microautophagy, and chaperone-dependent autophagy. The differences between particular types of autophagy result from the ways of supplying substrates for their degradation. One kind of macroautophagy, which involves mitochondrium sequestration, is called mitophagy. This process cleans the cell from damaged mitochondria, which are the source of proapoptotic factors (APAF-2, AIF, OMI/HTR2, SAMC/DIABLO) released to cell cytoplasm during apoptosis. Mitophagy is one of the mechanisms, which prevents mammary cancer cells from apoptosis caused by a long-lasting endoplasmic stress [38].

During ER stress the PERK kinase phosphorylates eIF2α and activates transcription factor ATF4 that participates in CHOP transcription. Both, ATF4 transcription factor and CHOP, bind with promotor areas of the genes vital for autophagy function. ATF4 activates the expression of *MAP1LC3B* gene, while CHOP is the activator of *ATG5* gene transcription. ATG5 protease participates in the transformation of cytosolic form of LC3-I of the molecular mass 18 kDa into a lipid-bound form of LC3-II of the molecular mass 16 kDa. LC3-II protein is integrated into the membrane of autophagosomes and its expression correlates with autophagy progression [39]. We demonstrated that low glucose concentration in the medium enhances the expression of CHOP transcription factor and concurrently activates autophagy in the breast cancer MCF-7 cell line.

The stage of autophagy initiation is most commonly regulated by mTOR protein kinase [36]. In cancer cells mTOR kinase participates in the growth, differentiation, proliferation, migration, survival, and control of metabolic processes in the cell [38, 39]. Malignant tumors of breast, ovaries, kidneys, large intestine, head, or neck are characterized by a constant, constitutive expression of mTOR kinase caused by a continuous stimulation of its activation pathway PI3 K/AKT/mTOR or mutations within coding genes [39, 40]. Our study confirms the hypothesis of the constant and constitutive expression of mTOR kinase in mammary cancer cells. We observed that the mTOR kinase expression is independent on glucose concentration in the medium and incubation time. It indicates that in the conditions of this experiment, autophagy was activated regardless of the changes in mTOR kinase expression. It seems that one of the possible ways of autophagy activation is the signal transduction pathway connected with the phosphorylation of P53 protein and P27 protein [36, 41].

**Fig. 9:**
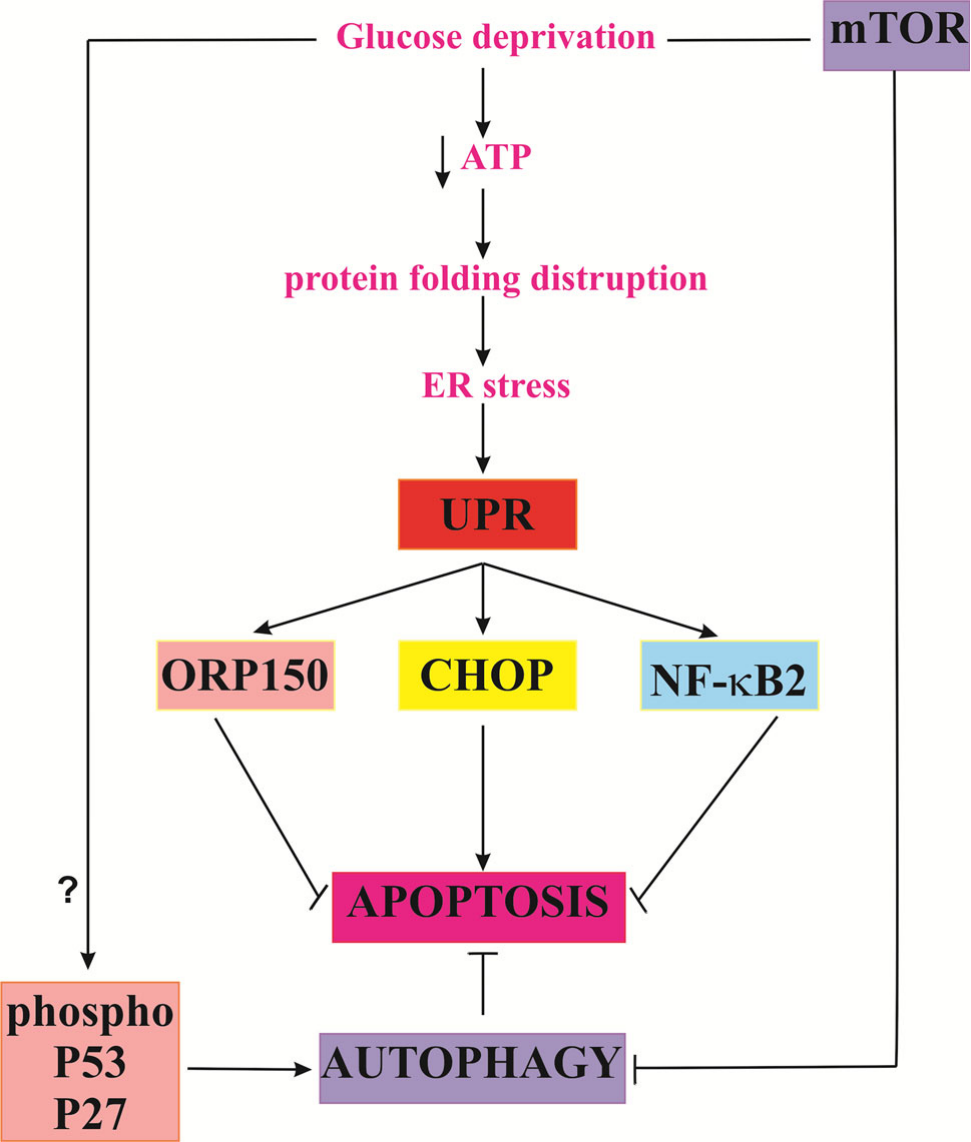
The effect of glucose shortage on apoptosis, autophagy, and their mechanism in breast cancer MCF-7 cell line. *ORP150* oxygen-regulated protein 150, *UPR* unfolded protein response, *NF-κB* nuclear factor-κB, *mTOR* mammalian target of rapamycin, ATP adenosine triphosphate, *CHOP* CCAAT/enhancer-binding protein (C/EBP) homologous protein, *P53* protein p53, *P27* protein p27

We suggest, that the ORP150 chaperone induction, transcription factor NF-κB2 activation, and increased autophagy protect breast cancer cells from apoptosis, despite the severity of the expression of proapoptotic transcription factor CHOP induced by a reduced glucose concentration in the medium (Fig. 9).

Recent studies have highlighted the importance of the coexistence of increased expression of ORP150 with decreased apoptosis in MCF-7 cells incubated in the medium with reduced concentration of glucose. It suggests that the glucose shortage, as a factor causing ER stress, induces the synthesis of ORP150, which protects cells from apoptosis. The explanation of both ORP150 function and its synthesis regulation provides new possibilities in the treatment of breast cancer. Nevertheless, for the first time we showed, that the induction of antiapoptotic chaperone ORP150, transcription factor NF-κB2 activation, and increased autophagy constitute protective mechanisms adapting MCF-7 cells to the stress derived from the endoplasmic reticulum stress. The observation, that glucose shortage may enhance resistance to apoptosis, has implication for the potential effects of this cancer treatment.
